# Acknowledgement to Reviewers of *Diagnostics* in 2018

**DOI:** 10.3390/diagnostics9010007

**Published:** 2019-01-09

**Authors:** Diagnostics Editorial Office

**Affiliations:** MDPI, St. Alban-Anlage 66, 4052 Basel, Switzerland

Rigorous peer-review is the corner-stone of high-quality academic publishing. The editorial team greatly appreciates the reviewers who contributed their knowledge and expertise to the journal’s editorial process over the past 12 months. In 2018, a total of 85 papers were published in the journal, with a median time to first decision of 17 days and a median time to publication of 36 days. The editors would like to express their sincere gratitude to the following reviewers for their cooperation and dedication in 2018:
Abuzaid, AhmedLoades, MariaAdhikari, PrakashLøbner-Olesen, AndersAhmed, AamirLodi, Matteo BrunoAlian, AymenLosurdo, GiuseppeArmstrong, GlenLum, Ying WeiAshish, PathakLyon, GholsonBanitsas, Konstantinos A.Ma, MichelleBauckneht, MatteoMainprize, JamesBechsgaard, Rie EriksenMargolies, LaurieBellani, MarcellaMarín, MercedesBender, Hans-UlrichMartins, Ian JamesBernardello, FabioMasarone, DanieleBernassau, AnneMavridis, KonstantinosBernemann, ChristofMayo, RayBharaj, PreetiMcmorrow, TaraBlyth, Benjamin J.Mejzlik, JanBoard, MaryMercado-Shekhar, Karla P.Bordonaro, MichaelMinaga, KosukeBrewer Gutierrez, OlayaMirza, Aleem KHCai, LishengMohd Shah, Syaiful RedzwanCarding, SimonMöhlendick, BirteCarpentieri, DavidMoncayo, RoyCasals Terre, JasminaMorton, BenCascini, Giuseppe LucioMoseley, TanyaCatry, BoudewijnMoxon, JosephCavagnaro, MartaNacul, Luis CChalah, MoussaNery, FabioChatterjee, JhinukNima, Zeid A. Chen, WenchunNorcic, GregorChoi, Yun-JungNyström, PärClancy, EoinOlivito, GiusyClauser, PaolaOmura, YasuhisaCortés Castell, ErnestoOsaki, TakakoCoskun, AhmetOzen, Mehmet OzgunDakhlallah, DuaaPage, Anne-LaureDaley, PeterPapacleovoulou, GeorgiaDavalieva, KatarinaParsons, Lauren N.Denomme, Gregory A.Pasian, MarcoDeshpande, ShayuPastey, ManojDi Donato, LoretoPerez, MauricioDiermeier, SarahPetersen, LarsDietrich, Johannes W.Popovic, MilicaDimitrakopoulou-Strauss, AntoniaPorter, EmilyDing, XuemeiPotus, FrançoisDragomir, MihneaPovlsen, SebastianDrukker, KarenQasaimeh, Mohammad A.Dunne, Eileen MQiu, HaoEboigbodin, KevinQiu, ZhenEichhorn, IngaRahib, LolaElshimali, Yahya I.Rahimi, FaridEmoto, MakotoRainger, George EdwardFang, XiaolanRamalingam, NaveenFedeli, AlessandroRamchandani, DivyaFerlizza, EneaReif, Kathryn E.Ferrer-Cascales, Rosario I.Ritter, KerstinFina, EmanuelaRojo-Álvarez, José LuisFlegel, Willy A.Rosenzweig, AllisonFloresta, GiuseppeRydosz, ArturGallerani, GiuliaSan Miguel, AdrianaGerke, OkeSankarasubramanian, VishwanathGjörloff Wingren, AnetteSaponara, SergioGo, Michael R.Saraste, AnttiGombert, AlexanderSathe, Gajanan JalindarnathGregory, AndrewSathekge, MikeGuan, WeihuaSato-Bigbee, CarmenGupta, RisheinSciacchitano, SalvatoreGupta, VijayalaxmiSerafini, GianlucaHamad-Schifferli, KimberliSerefko, AnnaHan, Seung HeeShah, JyotsnaHelbig, MarkoShao, DangdangHendrikse, HarryShi, JiahaiHingorani, DinaShiao, Young-JiHossain, Md. DelwarShim, Hyo SupHsieh, KuangwenSingh, VirtajHsu, Sung-PoSmith, Heather F.Hu, XiaosuSoares, PaulaHuffman, MarkSong, Yong-Ak (Rafael)Ierardi, EnzoSorensen, Karina DalsgaardIinuma, HisaeSpeight, NigelIslam, Md AsifulSperber, Geoffrey H.Isola, GaetanoSpizzo, GilbertJaiswal, Ashvin R.Sreerama, SubramanyaJason, FieringStaines, DonaldJeong, Keun-YeongStrand, Sven-ErikJeschke, UdoTaguchi, Y-h.Jiménez-Bonilla, Julio FranciscoTakayama, KenichiJoshi, BharatTan, IsabellaKairemo, KaleviTcheslavski, Gleb V.Kaushik, AjeetTeo, Soo KngKerr, JonathanTerracciano, DanielaKhoo, Bee LuanTheodorsson, ElvarKim, Nam-YoungTsukamoto, TetsuyaKlein, Katherine A.Twisk, FrankKnippa, EmilyUpadhyay, AbhinavKotsiantis, SotirisUwiera, TrinaKottner, JanVanderWeele, DavidKoutsoupidou, MariaVashist, ArtiKrishna, Somashekar G.Vasilevsky, NicoleKumar, SuneelVaughan, Christopher L.Kung, Yu-ChunVenkataraman, RajeshLandsheer, HansVijayaraghavan, Gopal R.Le Pichon, Jean-BaptisteVitorino, RuiLehmann-Chehe, JacquelineWei, ChangyongLenzo, NatWilczek, BrigitteLeon Mateos, LuisXia, HuiLewis, MarkXu, ZhihuoLi, LinYe, YanqiLi, Xiang-GuoYoussofzadeh, VahabLidbury, BrettZacho, Helle DLima, IvanZagidullin, NaufalLiu, Wei-MinZakaria, AmerLiu, YuZun, Zungho

